# Activation of TREK‐1 and TREK‐2 Two‐Pore Domain Potassium Channels by the K_v_4 Channel Modulator, NS5806

**DOI:** 10.1002/prp2.70264

**Published:** 2026-05-14

**Authors:** E. L. Veale, P. M. Matthews, A. M. Rush, E. B. Stevens, A. Mathie

**Affiliations:** ^1^ Medway School of Pharmacy University of Kent and University of Greenwich Chatham Maritime UK; ^2^ Metrion Biosciences Cambridge UK; ^3^ School of Life Sciences University of Westminster London UK

**Keywords:** K_V_4, negatively charged activators, NS5806, TREK, trigeminal neurons, two‐pore domain potassium channel

## Abstract

NS5806 is a diaryl‐urea small molecule developed for the treatment of pain and neurological disorders. A potent activator of K_v_4.3 potassium channels, it is widely used to study K_v_4 channel physiology in excitable cells, including trigeminal neurons. Surprisingly, we found that NS5806 produced a significant, robust hyperpolarization of the resting membrane potential of all trigeminal neurons tested. Given the structural similarity of NS5806 to other negatively charged activator compounds with broad potassium channel activity, we investigated whether NS5806 might also modulate the two‐pore domain potassium (K2P) TREK channels, which are abundantly expressed in sensory neurons. Here we show that NS5806 activates both TREK‐1 and TREK‐2 channels. While NS5806 is described as a K_v_4‐selective activator, our data suggest that its effects on trigeminal and other neurons may arise from concurrent activation of multiple potassium channel types, including TREK‐1 and TREK‐2.

## 1 Introduction

NS5806 is a diaryl‐urea small molecule, developed for the treatment of pain and neurological disorders of the central nervous system. In vitro, NS5806 has been identified as a potent activator of K_v_4‐family A‐type potassium channels, specifically K_v_4.3 via the accessory subunit KChIP2 [1, 2, 3, 4] and it is described as a K_v_4.3 specific activator. Consequently, NS5806 has become a key tool in investigating K_v_4 channel physiology and subunit composition in excitable cells, where K_v_4.1 and 4.3 are differentially expressed (e.g., [5]). Additionally, NS5806 has shown promise in its ability to attenuate neuropathic pain in animal models of injury‐induced K_v_4 channel downregulation [6].

Functionally, K_v_4 channels contribute to transient A‐type outward currents that regulate action potential repolarisation and firing frequency but have minimal influence on resting membrane potential (RMP) [7]. In this study, we investigated the effects of NS5806 on trigeminal neuron excitability. Surprisingly, this compound produced a significant, robust hyperpolarization of the resting membrane potential of all neurons tested.

Given that TREK‐1 and TREK‐2 two‐pore domain potassium (K2P) channels are highly expressed in small‐ and medium‐sized sensory neurons, where they play a critical role in maintaining the RMP and regulating neuronal excitability [8, 9], we hypothesized that, in addition to its known action on K_v_4.3 channels, NS5806 might also have an additional action modulating TREK channel activity.

## 2 Methods

### 2.1 Electrophysiological Recordings of Cultured Rat Trigeminal Neurons

Acutely dissociated rat trigeminal neurons were isolated from neonatal Wistar rats (P4‐12). Trigeminal ganglia were dissociated by mincing in Ca^2+^/Mg^2+^‐free HBSS followed by sequential enzymatic digestion with dispase II (2.5 mg/mL, 37°C, 30 min; Sigma, Poole, UK) and collagenase (1.0 mg/mL, 37°C, 30 min; Sigma, Poole, UK). Tissue was washed, gently triturated, filtered through a 40 μm strainer, and centrifuged (200*g*, 6 min). Cells were resuspended in complete medium and plated onto poly‐D‐lysine/laminin–coated coverslips and used for experiments from DIV0 to DIV2. Animal studies are reported in compliance with the ARRIVE guidelines. Cells were perfused with an extracellular solution containing (in mM): 135 NaCl, 4.7 KCl, 1 CaCl_2_, 1 MgCl_2_, 10 HEPES and 10 glucose, pH 7.4, with NaOH (310 mOsmol·L^−1^). Pipette solution contained (in mM): 130 KCl, 1 MgCl_2_, 5 MgATP, 0.3 NaGTP, 10 HEPES and 5 EGTA, pH adjusted to 7.3 with KOH (290 mOsmol·L^−1^). Manual patch‐clamp experiments were performed at room temperature (~21°C) using MultiClamp 700B (Axon Instruments, Molecular Devices) patch‐clamp amplifier using Clampex 10.7 (Axon Instruments, Molecular Devices) software. Glass patch pipettes were fabricated on an electrode puller from borosilicate glass capillaries (Harvard Apparatus) to resistances between 1.8 and 2.2 MΩ. Action potential firing and resting membrane potential (RMP) were recorded from small cells (< 30 pF). NS5806 was dissolved in 0.3% DMSO as 10 mM stock and applied in the vicinity of cells using a gravity fed perfusion system.

### 2.2 Electrophysiological Recordings of Human TREK Channels in tsA201 Cells

Whole‐cell patch‐clamp recordings were performed using tsA201 cells (a HEK293T derivative, ECACC; Merck, UK) transiently transfected with either human TREK‐1 (KCNK2, NM_014217, pcDNA3.1 (Invitrogen, UK)) or TREK‐2 (KCNK10, NM_138318), pCMV6‐XL4 (Origene, USA). Whole‐cell configuration was achieved by formation of a high‐resistance gigaseal followed by membrane rupture using gentle suction. cDNA (0.5 μg) was co‐transfected with a GFP‐expressing reporter plasmid using the calcium‐phosphate method. Cells were maintained at 37°C in 5% CO_2_ and used for experiments 18–24 h post‐transfection. Independent transfections and recordings were performed across multiple experimental sessions over an 8–12‐week period to ensure reproducibility. Recordings were made at room temperature (20°C–25°C) using an Axopatch 1D amplifier (Molecular Devices, Sunnyvale, CA), low‐pass filtered at 2 kHz, and digitized at 5 kHz via a Digidata interface in a voltage‐clamp configuration. Glass pipettes (GC150TF, Harvard Apparatus, Edenbridge, UK) were pulled to a resistance of 4–6 MΏ and filled with an internal solution containing (in mM): 150 KCl, 3 MgCl_2_, 5 EGTA and 10 HEPES (pH 7.4 with KOH). The extracellular bath solution contained (in mM): 145 NaCl, 2.5 KCl, 3 MgCl_2_, 1 CaCl_2_, and 10 mM HEPES (pH 7.4 with NaOH). All reagents were obtained from Merck (UK).

A range of steps and ramps in voltage were applied from a holding potential of −60 mV to assess current–voltage relationships. Outward current was quantified as the difference in current amplitude between −80 and −40 mV in order to minimize possible contribution from endogenous voltage‐gated potassium channels present in tsA201 cells. Data are presented as mean ± 95% confidence interval (CI), with *n* representing individual cells. NS5806 (N‐[3,5‐bis(trifluoromethyl)phenyl]‐N′‐[2,4‐dibromo‐6‐(1H‐tetrazol‐5‐yl)phenyl]‐urea, Merck UK) was prepared as a 10 mM stock in DMSO and diluted in extracellular solution immediately prior to use and applied via continuous gravity‐driven bath perfusion at (4–5 mL min^–1^) for 2–3 min until a steady‐state response was achieved. Washout was performed using control external solution for ≥ 5 min or until recovery was observed. Final DMSO concentration did not exceed 0.1% (v/v).

For all experiments, currents were analyzed using pCLAMP, Microsoft Excel, and GraphPad Prism.

## 3 Results

### 3.1 Effects of NS5806 on Capsaicin‐Sensitive and Retigabine‐Sensitive Trigeminal Neurons

**FIGURE 1:**
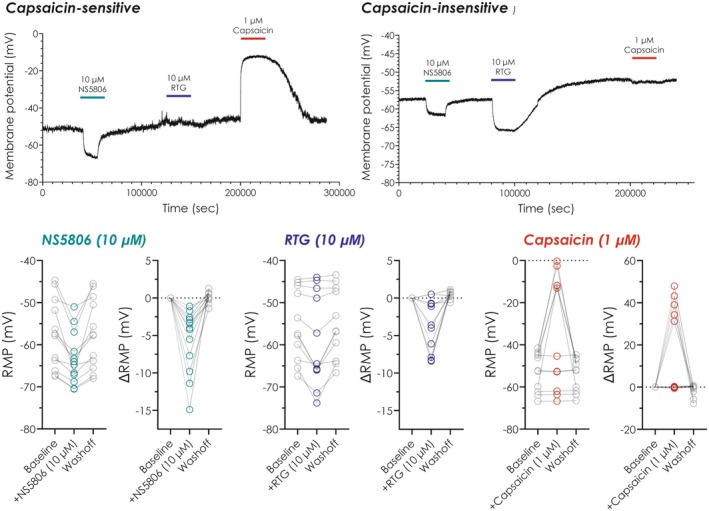
NS5806 induces hyperpolarization and reduces excitability in capsaicin and retigabine‐sensitive trigeminal neurons. Top: Representative current‐clamp recordings from a capsaicin‐sensitive trigeminal neuron (left hand side) and retigabine‐sensitive neuron (right hand side) showing membrane potential (mV) before and after application of NS5806 (10 μM), retigabine (10 μM) and capsaicin (1 μM). Bottom: Combined plots showing absolute and normalized RMP shifts (mV) in 12 cells in response to 10 μM NS5806 (left hand side, green), 10 μM retigabine (middle, blue) and 1 μM capsaicin (right hand side, red).

Application of NS5806 (10 μM) to trigeminal neurons produced a significant and reversible hyperpolarization of the RMP in all neurons tested (Figure 1). On average NS5806 shifted the RMP from −57.6 ± 2.4 mV (mean ± SEM, *n* = 12) under control conditions to −63.3 ± 1.8 mV (mean ± SEM, *n* = 12) during drug application and back to −57.4 ± 2.4 mV (mean ± SEM, *n* = 12) following washout of compound. This corresponded to a mean hyperpolarization of −5.7 ± 0.12 mV (*n* = 12, *p* < 0.01, one‐way ANOVA with Tukey's HSD). This hyperpolarising effect was observed consistently in both capsaicin‐sensitive/retigabine‐insensitive neurons where NS5806 shifted the RMP by −7.8 ± 2.7 mV (SEM, *n* = 5, *p* < 0.05, paired *t*‐test) and in capsaicin‐insensitive/retigabine‐sensitive neurons (−4.3 ± 0.7 mV, *n* = 7, *p* < 0.001, paired *t*‐test). There was no significant difference in the magnitude of hyperpolarization between the two groups (unpaired *t*‐test, *p* > 0.05) indicating that NS5806 acts independently of capsaicin or retigabine sensitivity, the latter suggesting it does not act on K_V_7 channels. Given the negligible contribution that K_V_4 channels have on neuronal resting membrane potential [7], this was a somewhat surprising observation and suggests that perhaps NS5806 acts broadly on trigeminal neuron populations to suppress excitability, potentially through other potassium channels expressed in trigeminal neurons and involved in modulating the RMP.

### 3.2 Structural Similarities Among NS Compounds

NS5806 exhibits strong structural similarity to other NS compounds, particularly NS3623 (1‐(3‐trifluoromethylphenyl)‐3‐[2‐(1H‐tetrazol‐5‐yl]‐4‐bromophenyl]urea) and NS11021 (1‐(3,5‐bis‐trifluoromethyl‐phenyl)‐3‐[4‐bromo‐2‐(1H‐tetrazol‐5‐yl)‐phenyl]‐thiourea). As shown in Table 1, all three compounds share a conserved negatively charged activator (NCA) pharmacophore, characterized by a hydrophobic CF_3_ moiety (yellow), an aromatic ring scaffold (blue), and a negatively charged tetrazole group (red), arranged at defined spatial distances. These recurring features are hallmarks of the NS series and are thought to underpin their ion channel modulatory activity, promoting channel opening at the selectivity filter. Consistent with previous reports describing the polypharmacology of NCAs [10], the close structural resemblance among these compounds is reflected in their broad activation profiles. Collectively, they modulate multiple K^+^ channel families including voltage‐gated hERG (K_v_11.1) channels, voltage‐gated potassium (K_v_4) channels, two‐pore domain potassium (K2P) channels, and calcium‐activated BK type channels (BK_Ca_).

**TABLE 1:** Structural features and targets of selected NS‐series compounds. Created with BioRender.com.

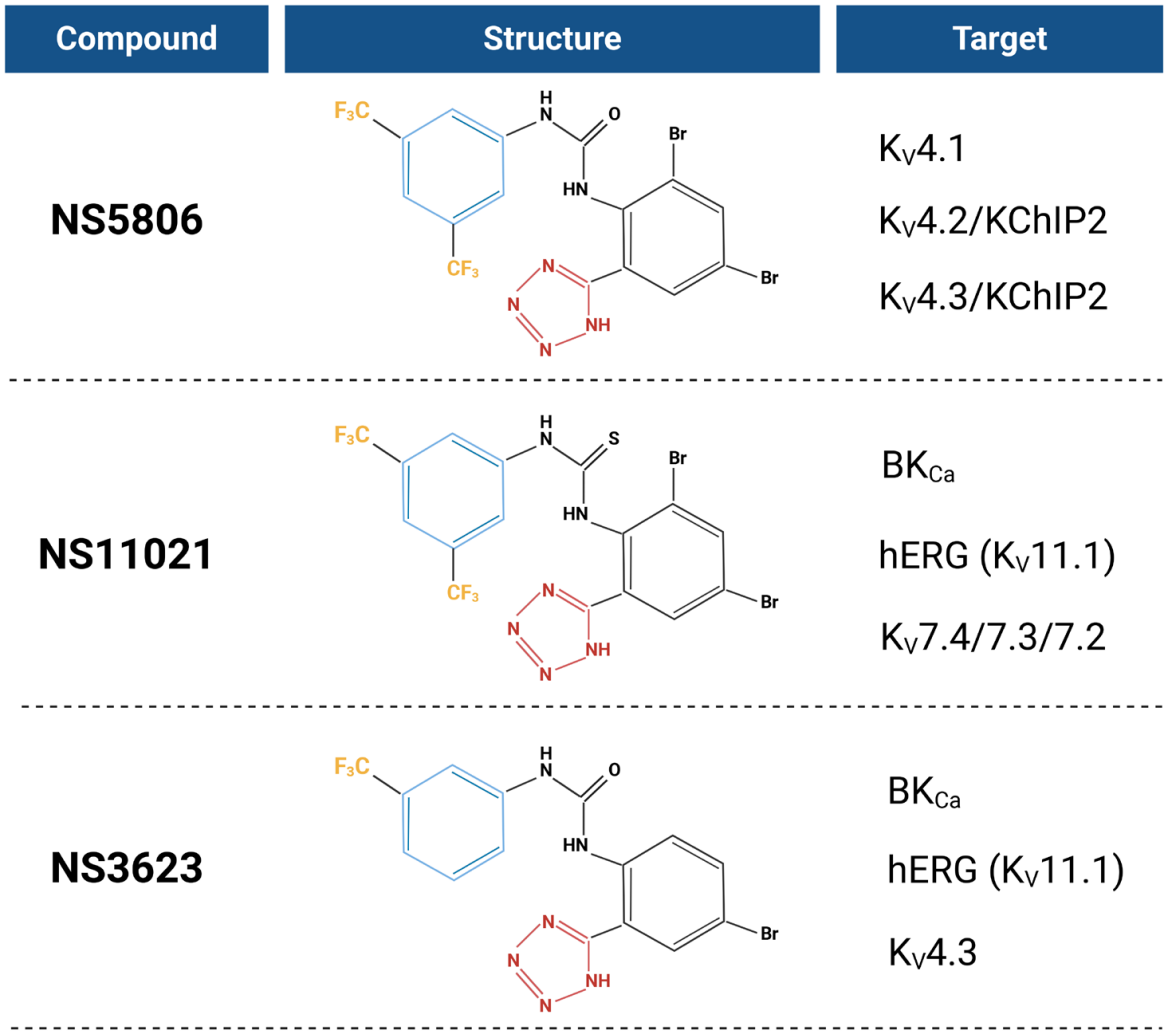

### 3.3 Activation of Recombinant TREK Channels by NS5806

**FIGURE 2:**
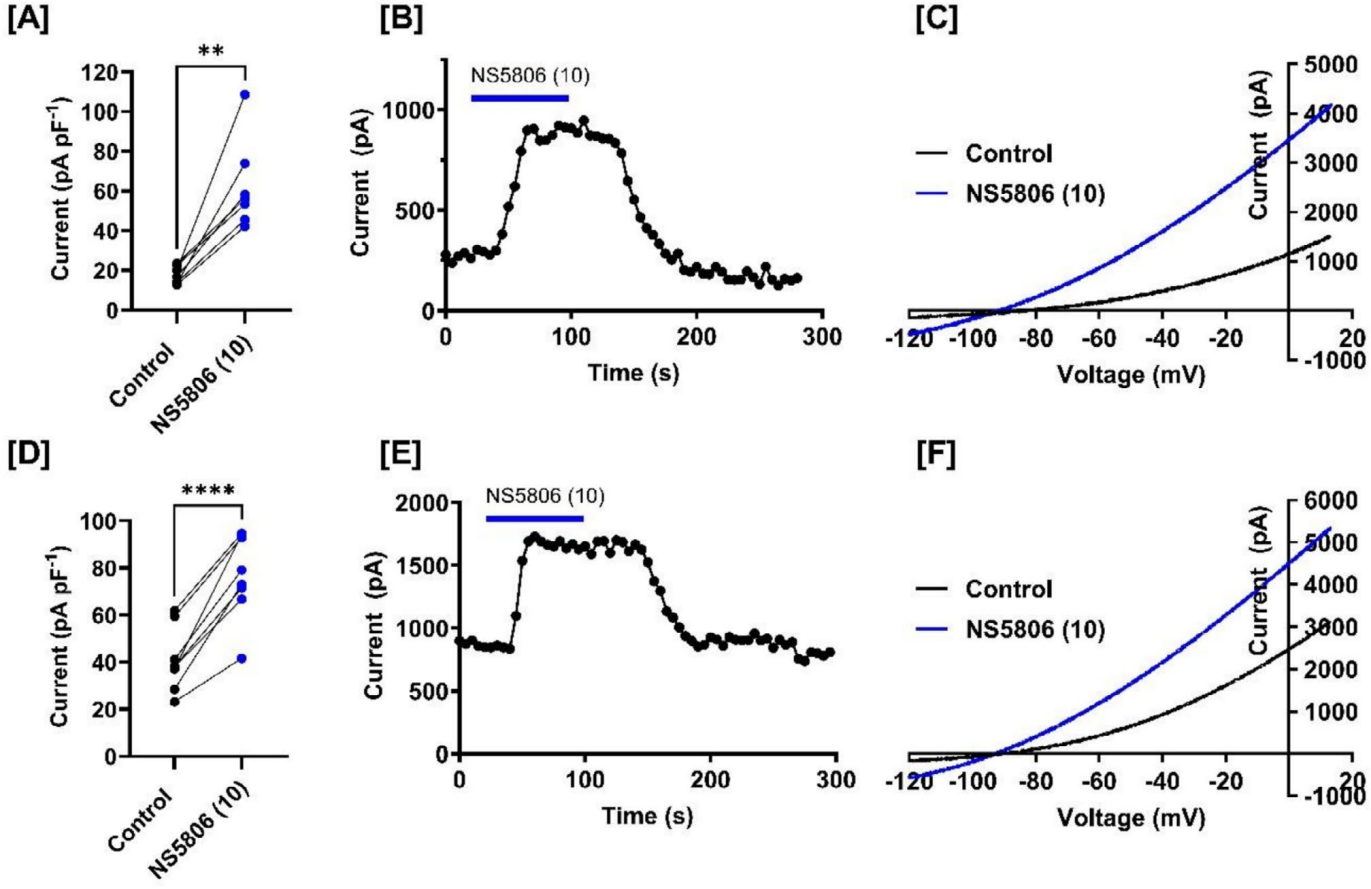
NS5806 enhances currents through TREK‐1 and TREK‐2 K2P channels. (A, D) Paired plots showing peak current density (pA pF‐1) for TREK‐1 (A) and TREK‐2 (D) in control (black dots) and following acute application of NS5806 (10 μM, blue dots). (B, E) representative time courses of whole‐cell current at −40 mV illustrating the rapid and reversible activation of TREK‐1 (B) and TREK‐2 (E) by NS5806. (C, F) current–voltage (I‐V) relationships for TREK‐1 (C) and TREK‐2 (F) in the absence (black trace) and presence (blue trace) of NS5806 (10 μM).

We hypothesized that, like other NS compounds, NS5806 might act as an activator of TREK (K2P) channels. To test this, we expressed TREK‐1 and TREK‐2 channels in tsA201 cells and measured whole‐cell currents in control solution and following acute application of NS5806 (10 μM). NS5806 (10 μM) significantly (*p* < 0.002, paired *t*‐test) enhanced TREK‐1 current from 18 pA pF‐1 (*n* = 7, 95% confidence intervals (CI):13–22), to 63 pA pF‐1 (*n* = 7, 95% CI: 41–84. Figure 2A), corresponding to a 261% increase (*n* = 7; 95% CI: 138–383) of control current. Similarly, TREK‐2 current was significantly increased from 41 pA pF‐1 (*n* = 8, 95% CI: 30–52) in control to 77 pA pF‐1 (*n* = 8, 95% CI: 62–92) in the presence of NS5806 (*p* < 0.0001, paired *t*‐test), equivalent to an average increase of 95% of the control current (95% CI: 61–128). The effect of NS5806 was reversible upon washout as illustrated in Figure 2B,E, showing representative recordings from single cells exposed to NS5806. Current–voltage (I‐V) relationships (Figure 2C,F) demonstrate that current amplitude increased across the voltage range tested with no obvious voltage dependence or alteration to the outwardly rectifying profile of TREK channel currents and no change in reversal potential. Together, these findings confirm our hypothesis that NS5806 is a potent activator of TREK channels.

## 4 Discussion

In this study, we show for the first time that the established K_v_4 activator NS5806 is also a potent activator of TREK K2P channels, significantly enhancing currents through both TREK‐1 and TREK‐2 channels. This expands the known pharmacological profile of NS5806 beyond its canonical K_v_4.3/KChIP2 activity and suggests that the compound may exert polypharmacological actions on multiple K^+^ channel families in sensory neurons. This raises the possibility that some prior interpretations of NS5806 effects may have overlooked a concurrent contribution from TREK channels or other K^+^ channel subtypes.

The structural similarities among NS5806, NS3623, and NS11021 compounds, known to activate diverse K^+^ channels including hERG, BK, and K2P families, support a broader pharmacological spectrum mediated by a conserved negatively charged activator (NCA) pharmacophore. These CF_3_ and tetrazole moieties appear central to channel interaction, suggesting that NS5806 engages TREK channels via similar binding determinants to the other NS compounds [10, 11].

Physiologically, dual activation of K_v_4 and TREK channels would be expected to stabilize RMP and reduce excitability synergistically, potentially explaining the robust hyperpolarization observed in our trigeminal neuron experiments. While such multi‐channel modulation could be therapeutically beneficial in conditions such as neuropathic pain, it complicates the mechanistic interpretation of NS5806‐based studies. Further work is needed to delineate the relative contributions of K_v_4 and K2P channels in sensory neuron excitability and to identify structural modifications that confer selectivity within the NS compound series. A deeper understanding of these interactions may aid in the design of new, more selective activators with therapeutic potential.

## 5 Conclusion

Our findings demonstrate that NS5806, whilst originally characterized as a K_v_4‐selective activator, also potently activates TREK‐1 and TREK‐2 K2P channels, thereby highlighting a broader spectrum of ion channel targets for the compound.

## Author Contributions

**E. L. Veale:** conceptualization, investigation, methodology, formal analysis, data curation, funding acquisition, project administration, writing – original draft, writing – review and editing. **A. Mathie:** conceptualization, methodology, formal analysis, writing – original draft, writing – review and editing. **P. M. Matthews:** investigation, formal analysis, writing – review and editing. **E. B. Stevens:** conceptualization, methodology, formal analysis, data curation, supervision, project administration, writing – original draft, writing – review and editing. **A. M. Rush:** investigation, formal analysis, supervision, project administration, writing – review and editing.

## Funding

E.L. Veale was supported by a Royal Society Industry Fellowship (IF\R1\251048) sponsored by Metrion Biosciences Ltd.

## Ethics Statement

Experiments involving cultured trigeminal neurons were conducted independently by Metrion, operating under UK Home Office licensing and institutional ethical approvals at Babraham Institute, Cambridge. Metrion sourced and used the neurons in full accordance with the Animals (Scientific Procedures) Act 1986 and the principles of the 3Rs (Replacement, Reduction, and Refinement). Animals were euthanised in accordance with Schedule 1 of the Act. No live animal work or primary tissue handling was conducted at either institute.

## Conflicts of Interest

The authors declare no conflicts of interest.

## Data Availability

The data that support the findings of this study are available from the corresponding author upon reasonable request.
